# Relationship Between Nutritional Status and Systemic Immune–Inflammation Indices Across BMI Categories

**DOI:** 10.3390/nu17233799

**Published:** 2025-12-03

**Authors:** Hafize Uzun, Aysen Kutan Fenercioglu, Abdulhalim Senyigit, Gunay Can

**Affiliations:** 1 Department of Medical Biochemistry, Faculty of Medicine, Istanbul Atlas University, 34403 Istanbul, Turkey; huzun59@hotmail.com; 2 Department of Family Medicine, Cerrahpasa Medical Faculty, Istanbul University-Cerrahpasa, 34098 Istanbul, Turkey; 3 Department of Internal Medicine, Faculty of Medicine, Istanbul Atlas University, 34403 Istanbul, Turkey; abdulhalim.senyigit@atlas.edu.tr; 4 Department of Public Health, Cerrahpasa Medical Faculty, Istanbul University-Cerrahpasa, 34098 Istanbul, Turkey; gunaycan09@yahoo.fr

**Keywords:** BMI, HALP score, PNI, NRI, CONUT, SII, NLR, PLR

## Abstract

**Background:** This study aimed to determine the relationship between nutritional status and systemic inflammation using four validated nutrition indices—Hemoglobin, Albumin, Lymphocyte, and Platelet (HALP) score, Prognostic Nutritional Index (PNI), Controlling Nutritional Status (CONUT) score, and Nutritional Risk Index (NRI)—and three immune–inflammation biomarkers—Systemic Immune–Inflammation Index (SII), neutrophil-to-lymphocyte ratio (NLR), and platelet-to-lymphocyte ratio (PLR)—in healthy adults with varying body mass index (BMI) levels. **Methods:** This retrospective study included 290 clinically healthy adults aged 18–60 years, categorized by BMI. Individuals with chronic diseases, medication use, or morbid obesity (BMI ≥ 40 kg/m^2^) were excluded. Nutrition scores (HALP, PNI, NRI, CONUT) and systemic immune–inflammation indices (SII, NLR, PLR) were calculated from laboratory data. For the comparisons of SII, PLR, NLR, PNI, HALP, NRI, and CONUT values between groups, age was adjusted for, and an ANCOVA test was performed. **Results:** Among the systemic immune–inflammation indices, SII and NLR were significantly higher in both the overweight and obesity groups. The CONUT score, a negative indicator of nutritional status, demonstrated positive correlations with SII, NLR, and PLR in the overweight group, and with PLR in the obesity group. Although PNI showed significant inverse correlations with SII, PLR, and NLR in both groups, the mean PNI values remained above 50, indicating overall normal nutritional status in the study population. HALP was inversely correlated with SII, PLR, and NLR in both groups. **Conclusions:** The HALP score appears to be the most reliable marker, as it reflects the inverse relationship between nutritional status and systemic immune–inflammation indices.

## 1. Introduction

Maintaining a healthy body mass index (BMI) is crucial for preventing premature death from high blood pressure, cardiovascular disorders, cerebrovascular diseases, and certain cancers. Overweight individuals or those with obesity are also at an increased risk of other chronic diseases such as type 2 diabetes, psychiatric disorders, respiratory diseases, and skeletal disorders [1,2,3]. A high BMI is associated with chronic low-grade inflammation. Excess adipose tissue promotes the secretion of pro-inflammatory cytokines, including tumor necrosis factor-α (TNF-α) and interleukin-6 (IL-6). This leads to macrophage infiltration into adipose tissue and decreases adiponectin synthesis, creating a proinflammatory environment and oxidative stress. These inflammatory factors can trigger a systemic inflammatory response, leading to diseases associated with obesity [1,3].

A complete blood count (CBC) is an affordable and easily accessible blood test. Biomarkers like the neutrophil-to-lymphocyte ratio (NLR), platelet-to-lymphocyte ratio (PLR), and Systemic Immune–Inflammation Index (SII) are derived from CBC and have been shown to help in the diagnosis, monitoring, and assessment of various systemic inflammatory conditions [1,2,3,4,5]. Neutrophils and lymphocytes are key players in inflammation. A lower NLR is linked to better outcomes in different cancer types and coronary artery disease [4,5,6,7,8]. In a Spanish study, an inflammatory state measured by NLR was positively associated with abdominal obesity and negatively associated with diet quality [9]. Another study conducted in adolescents with obesity found that NLR and *C*-reactive protein (CRP) levels were significantly higher in the obesity group compared to healthy controls [10]. Blood platelets also considerably contribute to the immune response in chronic inflammation caused by obesity [11]. The SII measures the relationship between platelet count, neutrophil count, and lymphocyte count and is considered the most effective tool to reflect the balance between inflammatory factors and immune responses in the body [1,2,3,4,5]. Studies have demonstrated a significant positive correlation between SII and obesity [1,2].

Malnutrition is often perceived to affect only underweight individuals. In fact, there is a large burden of malnutrition in the overweight and individuals with obesity due to inadequate micronutrient consumption and poor food quality [12]. Another reason for malnutrition in obesity is the inability to preserve body composition and performance due to sarcopenia [13]. The quality of diet affects systemic inflammation status in obesity [9]. Therefore, beyond BMI alone, routine nutritional screening is recommended for the management of most acute and chronic conditions, particularly in hospitalized patients. A validated and widely utilized nutritional screening tool includes the Controlling Nutritional Status (CONUT) score, the Nutritional Risk Index (NRI), and the Prognostic Nutritional Index (PNI). This is a nutritional assessment tool based on easily accessible laboratory indicators. Its superior prognostic value has been demonstrated [14]. Another scoring system for assessing a patient’s nutritional status and prognosis is the Hemoglobin, Albumin, Lymphocyte, and Platelet (HALP) score. The HALP score has recently emerged as a novel biomarker that reflects both nutritional reserves and immune–inflammatory activity [15,16].

The present study aimed to investigate the association between nutritional status and systemic inflammation according to BMI categories in a healthy population. For this purpose, individuals with normal and high BMI were evaluated for their nutritional status using HALP, PNI, CONUT, and NRI scores. The relationship between these nutritional indices and systemic immune–inflammation markers (SII, NLR, PLR) was then examined separately in normal- and high-BMI groups.

## 2. Materials and Methods

### 2.1. Ethical Approval

This study was conducted as a retrospective case–control study at the Department of Internal Medicine, Istanbul Atlas University Medical Faculty Hospital. The study was designed in accordance with the Declaration of Helsinki and good clinical practice guidelines. It was approved by Istanbul Atlas University Non-Interventional Scientific Research Ethics Committee (Approval number: E-22686390-050.99-61546, date: 17 February 2025). The Ethics Committee waived the requirement for written informed consent due to the retrospective design of the study.

### 2.2. Study Design

This retrospective study included individuals who attended the Internal Medicine Outpatient Clinic of Istanbul Atlas University for their annual health check-up between March and July 2024. During this period, a total of 290 healthy individuals were evaluated, of whom 156 had a normal body mass index, 71 were classified as overweight, and 63 were classified as individuals with obesity. Individuals with morbid obesity (BMI ≥ 40.0 kg/m^2^) were excluded from the study, as systemic inflammation is commonly present in this population and could confound the results. According to the WHO classification, individuals were categorized as normal weight with a BMI of 18.5–24.9 kg/m^2^, overweight with a BMI of 25.0–29.9 kg/m^2^, and obesity with a BMI ≥ 30.0 kg/m^2^. Individuals with a BMI ≥ 40.0 kg/m^2^ were classified as morbid obesity, corresponding to WHO Obesity Class III [17]. Individuals with normal BMI constituted the control group, while individuals who were overweight or with obesity constituted the study group.

The study included individuals between the ages of 18–60 who are completely healthy, do not use any medication that could affect inflammation, and do not smoke or consume alcohol. The sociodemographic characteristics of the participants, as well as their height, weight, waist circumference, physical examination findings, and laboratory test results, were obtained retrospectively from their medical records.

#### 2.2.1. Inclusion Criteria

Individuals aged 18 to 60 years who did not have a known chronic illness, did not use any medication that could affect inflammation, did not smoke, or consume alcohol were included in the study. Only those with a BMI between 18.5 and 39.99 kg/m^2^ were included in the study.

#### 2.2.2. Exclusion Criteria

Individuals with any chronic illness, such as diabetes, chronic renal insufficiency, thyroid dysfunction, hepatic dysfunction, metabolic syndrome, autoimmune disease, malignancy, active infection, or a recent history of trauma or surgery, were excluded from the study. Additional exclusion criteria included the use of steroids, anti-inflammatory drugs, antiepileptic medications, antioxidant supplements, or vitamin preparations; smoking or alcohol consumption; and pregnancy. Furthermore, individuals over the age of 60 and with a BMI ≥ 40.0 kg/m^2^ were excluded, as systemic inflammation is commonly present in this population and could confound the results.

### 2.3. Assessments

Comprehensive medical histories—including information on smoking and alcohol use, physical examination findings such as weight, height, and waist circumference, and laboratory test results—were obtained from the medical records of the subjects. Waist circumference was measured with the participant standing upright, feet shoulder-width apart, arms relaxed at the sides, and breathing normally. A non-stretchable, flexible measuring tape was used to measure the waist circumference at the midpoint between the lower margin of the last palpable rib and the top of the iliac crest. Body weight was measured with a margin of error of 0.1 kg using an electronic scale (Seca digital scale, 0.1 precision, Hamburg, Germany), allowing only underwear to remain. Height was measured with a Harpenden stadiometer (Seca mod. 240 ce 0123, made in Hamburg, Germany) with an error margin of 0.1 cm. Height measurements were performed in the vertical position with bare feet, feet together and parallel, and the shoulder and gluteal region in contact with the wall. BMI was calculated by dividing weight (kg) by height squared (m^2^).

Fasting venous blood samples were drawn between 8 and 10 a.m. after the subjects fasted overnight (10–12 h). Blood samples were drawn from the brachial veins in the brachial fossa and placed into plain tubes and anticoagulant-free tubes. The samples were centrifuged for 10 min at 4000× *g* rpm at 4 °C. Biochemical tests were performed immediately. The result of CBC was recorded with an automatic hematology analyzer (Sysmex, Sysmex XN-1000, Norderstedt, Germany).

Biochemical parameters such as fasting blood glucose (FBG), low-density lipoprotein cholesterol (LDL-C), very-low-density lipoprotein cholesterol (VLDL-C), high-density lipoprotein cholesterol (HDL-C), triglyceride, total cholesterol, total protein, albumin, uric acid, and creatinine were determined using enzymatic methods (Architect I2000, Abbott Park, IL, USA). Insulin levels were measured by the electrochemiluminescence immunoassay (ECLIA) method on Roche-Hitachi E170 (Roche/Hitachi MODULAR Analytics Combination Systems, Roche Diagnostics, Indianapolis, IN, USA). Hemoglobin A1c (HbA1c) determination was based on High-Performance Liquid Chromatography (HPLC) (Variant Turbo II, Bio-Rad Laboratories, Inc., Hercules, CA, USA). Homeostasis model assessment for insulin resistance (HOMA-IR) was calculated by using the following formula:

HOMA-IR = Fasting glucose (mg/dL) × Fasting insulin (mU/L)/405

#### Nutrition Scores

Nutritional indices, including the PNI score, CONUT score, and NRI score, and systemic immune–inflammation indices were calculated and interpreted based on established formulas from the literature [14,15,16,17,18,19,20].

The **PNI score** was calculated using the following formula:

**PNI** = (10 × serum albumin [g/dL]) + (0.005 × peripheral lymphocyte count [/mm^3^])

The Prognostic Nutritional Index (PNI) is a clinical tool originally developed to assess nutritional and immunological status, particularly in patients undergoing surgery or with chronic illnesses such as cancer, cardiovascular disease, and chronic inflammatory conditions. Lower PNI scores indicate a higher risk of malnutrition. A PNI score > 50 indicates normal nutritional and immune status, whereas scores between 45 and 50 suggest a mild risk of malnutrition, 40–45 indicate moderate risk, and scores < 40 reflect severe malnutrition or a high nutritional risk [18].

The **CONUT score** was determined using serum albumin level, total lymphocyte count, and total cholesterol level. The scoring system ranges from 0 to 12, with higher scores indicating worse nutritional status. The point allocation for each parameter is as follows:

Serum albumin (g/dL):

≥3.5 = 0 points; 3.0–3.49 = 2 points; 2.5–2.99 = 4 points; <2.5 = 6 points.

Lymphocyte count (/µL):

≥1600 = 0 points; 1200–1599 = 1 point; 800–1199 = 2 points; <800 = 3 points.

Total cholesterol (mg/dL):

≥180 = 0 points; 140–179 = 1 point; 100–139 = 2 points; <100 = 3 points.

Higher CONUT scores indicate nutritional deficiency and immunosuppression. The total CONUT score ranges from 0 to 12 and is interpreted as follows: 0–1 point: normal nutritional status, 2–4 points: mild risk of malnutrition, 5–8 points: moderate malnutrition, 9–12 points: severe malnutrition [19].

The **NRI score** was calculated using the formula:

**NRI** = (1.519 × serum albumin [g/dL]) + (41.7 × actual body weight [kg]/ideal body weight [kg])

Lower NRI scores indicate a higher risk of malnutrition. An NRI score of 100 or higher is considered indicative of the absence of nutritional risk. An NRI score between 97.5 and 99.9 suggests mild risk, 83.5 to 97.4 suggests moderate risk, and below 83.5 indicates severe risk of malnutrition [20].

The **HALP score** was calculated using the following formula:

**HALP** = [hemoglobin (g/L) × albumin (g/L) × lymphocyte count (/L)]/platelet count (/L)

This composite index reflects the interplay between nutritional and inflammatory status [15,16]. There is no universally accepted cut-off, as HALP values may vary by population and disease context. However, lower HALP scores suggest malnutrition, inflammation, or poor prognosis

Systemic Immune–Inflammation Indices:

SII is a new and integrated inflammatory biomarker derived from CBC parameters that reflects the balance between the host’s immune and inflammatory status. SII was calculated using the formula [1]:

**SII** = (platelet count [/L] × neutrophil count [/L])/lymphocyte count [/L]

The SII index reflects both innate (neutrophils, platelets) and adaptive (lymphocytes) immune responses.

In addition to SII, NLR and PLR were also calculated for each subject using their respective neutrophil, lymphocyte, and platelet counts obtained from the CBC.

Neutrophil-to-Lymphocyte Ratio (NLR) and Platelet-to-Lymphocyte Ratio (PLR) are calculated using the following formulas [9,11]:

**NLR** = absolute neutrophil count [/L])/absolute lymphocyte count [/L]

**PLR** = platelet count [/L]/absolute lymphocyte count [/L]

### 2.4. Statistical Analysis

The statistical analyses were performed using SPSS version 27 (Statistical Package for the Social Sciences, IBM Corp., Armonk, NY, USA). Quantitative variables were presented as mean, standard deviation, median, minimum, and maximum values, while qualitative variables were expressed as frequency and percentage. The normality of data distribution was assessed using the Shapiro–Wilk test, skewness and kurtosis values, and Box Plot graphics.

For the comparison of more than two normally distributed quantitative variables, the One-Way ANOVA test was applied, and the Bonferroni test was used for post hoc pairwise comparisons. For non-normally distributed variables, the Kruskal–Wallis test was used, and the Dunn test was applied to identify the source of the difference (Table 1).

For the comparisons of SII, PLR, NLR, PNI, HALP, NRI, and CONUT values between groups, age-adjusted ANCOVA analyses were conducted. Bonferroni post hoc tests were subsequently applied to identify the specific groups contributing to the observed differences (Table 2).

To evaluate the relationships between variables, partial correlation analyses were performed for SII, PLR, NLR, PNI, HALP, NRI, and CONUT values, while adjusting for the effect of age (Table 3 and Table 4).

## 3. Results

The study population was divided into three groups based on BMI: normal weight (n = 156), overweight (n = 71), and obesity (n = 63). A statistically significant difference in mean age was observed across the groups, with individuals in the obesity group being older on average (*p* = 0.001). Although these differences were statistically significant, the observed age ranges suggest that they are unlikely to be clinically meaningful. Moreover, age-adjusted covariance analyses for the relevant parameters did not alter the level of statistical significance, indicating that age did not materially influence the results. As expected, waist circumference increased progressively with BMI and was significantly higher among participants with obesity (*p* = 0.001) (Table 1).

### 3.1. Comparison of Metabolic Parameters Across BMI Categories

Among the inflammatory and metabolic parameters, CRP, FBG, HbA1c, insulin, HOMA-IR, urea, uric acid, total cholesterol, triglyceride, VLDL-C, HDL-C, and LDL-C levels showed significant differences across BMI groups. In particular, differences in CRP, insulin, and HOMA-IR levels were significantly elevated in the obesity group, indicating a potential increase in systemic inflammation and insulin resistance (*p* = 0.001) (Table 1). No significant differences were observed for white blood cell (WBC) or creatinine levels (Table 1).

### 3.2. Relationship Between Nutrition Scores and Systemic Immune–Inflammatory Indices Across BMI Categories

**Figure 1:**
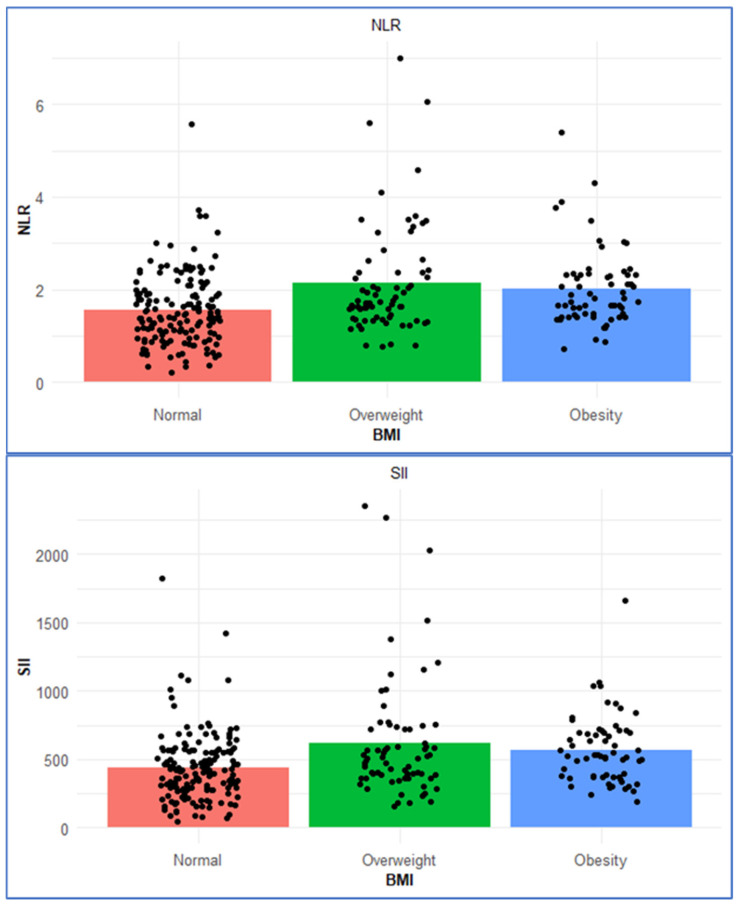
SII and NLR across BMI Categories.

Table 2 shows the association of BMI with nutrition scores and systemic immune–inflammatory indices. Although the NRI increased significantly with BMI (*p* = 0.001), it remained above 100 in all BMI categories, indicating no apparent nutritional risk across groups. No significant differences were found in PNI scores among the groups (*p* = 0.18). The mean CONUT scores in all BMI groups were <1, indicating a normal nutritional status across the study population. Similarly, the PNI scores were >50 in all groups, indicating a normal nutritional status across the study population (Table 2). There were no statistically significant differences in HALP scores among the BMI groups (*p* = 0.261). Regarding systemic immune–inflammatory indices, SII and NLR were significantly higher in the obesity and overweight groups compared to normal-weight individuals (*p* = 0.001 for both), suggesting heightened inflammatory activity. There were no statistically significant differences observed in PLR (*p* = 0.16) (Table 2). Figure 1 demonstrates the distribution of SII and NLR across BMI categories.

### 3.3. Correlation of Nutrition Scores with Systemic Immune–Inflammatory Indices in High BMI Groups

In overweight individuals, significant positive correlations were observed among the systemic inflammatory indices. Strong positive correlations were found between SII and PLR (r = 0.837; *p* < 0.001) and between SII and NLR (r = 0.844; *p* < 0.001). SII showed significant negative correlations with PNI (r = −0.295; *p* = 0.013) and the HALP score (r = −0.23; *p* = 0.046). Additionally, SII demonstrated a significant positive correlation with CONUT, a negative indicator of nutritional status (r = 0.307; *p* = 0.01) (Table 3).

Strong negative correlations were found between PLR and PNI (r = −0.392; *p* = 0.001) and between PLR and HALP (r = −0.307; *p* = 0.01). No significant correlation was observed between PLR and NRI. PLR showed a statistically significant positive correlation with CONUT (r = 0.302; *p* = 0.011) (Table 3).

No significant correlations were observed between NLR and HALP or NRI. A statistically significant positive correlation was observed between NLR and CONUT (r = 0.449; *p* < 0.001), and a strong negative correlation was observed between NLR and PNI (r = 0.462; *p* < 0.001) (Table 3).

Among the nutritional indices, PNI showed a positive correlation with HALP (r = 0.342, *p* = 0.004). A statistically significant negative correlation was found between PNI and CONUT (r = −0.292; *p* = 0.014) (Table 3).

In participants with obesity, strong positive correlations were observed among systemic inflammatory indices. SII showed strong positive correlations with PLR (r = 0.6, *p* < 0.001) and NLR (r = 0.887, *p* < 0.001). PLR was also positively correlated with NLR (r = 0.371, *p* = 0.003). SII correlated negatively with HALP (r = −0.522, *p* < 0.001). No significant correlations were observed between SII and NRI or CONUT (Table 4).

A strong positive correlation was observed between PLR and NLR (r = 0.371; *p* = 0.003). PLR also showed significant negative correlations with PNI (r = −0.363, *p* = 0.004) and HALP (r = −0.809, *p* < 0.001). No statistically significant correlation was observed between PLR and NRI, while a strong positive correlation was found between PLR and the CONUT score (r = 0.378; *p* = 0.002) (Table 3).

NLR was inversely correlated with PNI (r = −0.498, *p* < 0.001) and HALP (r = −0.358, *p* = 0.004). No statistically significant correlation was found between NLR and the CONUT score, while a significant negative correlation was observed between NLR and NRI (r = −0.299; *p* = 0.018) (Table 4).

Among the nutritional indices, PNI demonstrated a strong positive correlation with HALP (r = 0.601, *p* < 0.001). No statistically significant correlation was found between PNI and NRI or CONUT. A significant negative correlation was observed between HALP and NRI (r = −0.256; *p* = 0.045). The CONUT score did not show significant correlations with any of the inflammatory or nutritional indices (all *p* > 0.05) (Table 4).

These findings indicate that higher inflammatory burden (particularly SII, PLR, and NLR) is generally associated with poorer nutritional indices, whereas better nutritional status tends to accompany reduced systemic inflammation.

## 4. Discussion

**Figure 2:**
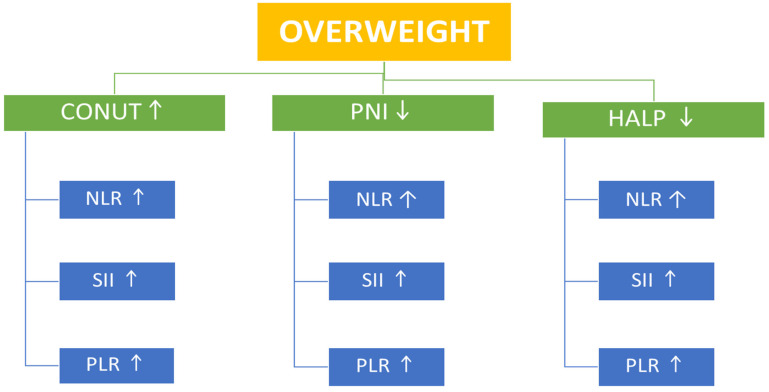
Associations of Nutritional Indices with Systemic Immune–Inflammatory Markers in the Overweight Group. ↑ = increase, ↓ = decrease.

**Figure 3:**
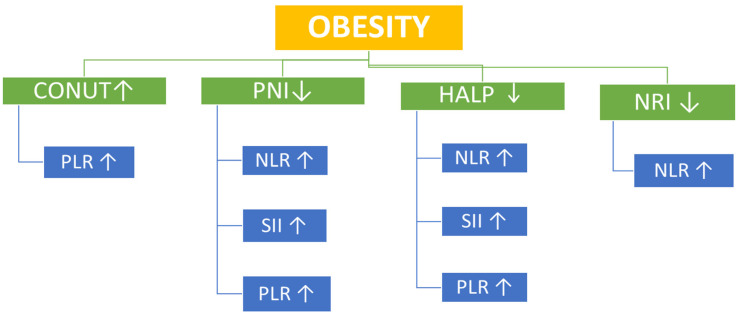
Associations of Nutritional Indices with Systemic Immune–Inflammatory Markers in the Obesity Group. ↑ = increase, ↓ = decrease.

This study examined the relationship between nutritional status and systemic immune–inflammatory indices across BMI categories in an adult population without any chronic diseases. Our findings show that as BMI increases, there is a corresponding rise in inflammation-related biomarkers (e.g., CRP, SII, NLR) (Table 1 and Table 2). Regarding nutritional scores, although the NRI score increased significantly with BMI (*p* = 0.001), it remained ≥100 across all BMI categories, indicating no apparent nutritional risk in any group. No significant differences were found in PNI or HALP scores among the groups (Table 2). The mean CONUT scores in all BMI groups were <1, indicating a normal nutritional status across the study population. Similarly, the PNI scores were >50 in all groups, indicating a normal nutritional status across the study population (Table 2). However, correlational analysis provided additional insights. In overweight participants, the CONUT score—a negative indicator of nutritional status—showed significant positive correlations with SII, PLR, and NLR, whereas PNI and HALP scores demonstrated significant inverse correlations with SII, PLR, and NLR. In the obesity group, the CONUT positively correlated with PLR, while PNI and HALP scores inversely correlated with SII, PLR, and NLR (Table 3 and Table 4) (Figure 2 and Figure 3).

These results support the growing body of evidence suggesting that adiposity contributes to a persistent low-grade inflammatory state [1,2]. Adipose tissue functions not only as a reservoir for energy storage but also as an immune-active organ, secreting a range of pro-inflammatory mediators collectively referred to as adipokines, including adiponectin, leptin, tumor necrosis factor-alpha (TNF-α), interleukin (IL)-1β, and IL-6 [21]. Dysregulation of adipokine production contributes to the establishment of a chronic inflammatory milieu within adipose tissue. As adipocytes undergo hypertrophy and lipid accumulation increases, immune cell infiltration—particularly by macrophages—intensifies, thereby amplifying both local and systemic inflammatory responses. In addition to macrophages, other immune and hematologic cells—including neutrophils, platelets, and lymphocytes—contribute significantly to the chronic low-grade inflammation associated with obesity [22,23]. Neutrophils are among the earliest immune cells to infiltrate adipose tissue in early obesity, releasing inflammatory mediators that amplify macrophage recruitment and maintain a chronic inflammatory state, thereby contributing to increased NLR and SII levels [24]. Neutrophils exacerbate both local and systemic inflammation by releasing pro-inflammatory mediators such as TNF-α and IL-1β. Platelets facilitate immune cell recruitment by secreting a variety of cytokines and chemokines. thereby amplifying the inflammatory response. Meanwhile. adaptive immunity is also altered: obesity induces a decline in regulatory T-cells and an increase in effector T-cell activity, shifting the balance toward pro-inflammatory T-cell subsets and further amplifying chronic low-grade inflammation—all of which may be captured by markers like PLR [25].

Zhang et al. demonstrated a U-shaped relationship between the systemic immune–inflammation index (SII) and adolescent obesity, suggesting that both low and high SII levels may be associated with an increased risk of obesity in the population [1]. In contrast, Yu et al. reported a significant positive correlation between SII and body mass index (BMI), particularly among adults under 60 years of age who did not have hypertension or diabetes [2]. Similarly, in our study population, which included individuals aged 18–60 years without any chronic disease, SII was found to increase significantly with rising BMI (*p* = 0.001).

Consistent with previous research, individuals with obesity in our study exhibited significantly higher levels of CRP, insulin, and triglycerides (Table 1), all of which are established markers of metabolic dysfunction and inflammation [9,22]. Although BMI is the conventional metric for classifying obesity, waist circumference also increases significantly with BMI and may provide additional insight into visceral adiposity and cardiometabolic risk [4,9]. In our study, waist circumference was significantly elevated in both the overweight and obesity groups (*p* = 0.001). Rodríguez-Rodríguez et al. reported that abdominal obesity was associated with a heightened inflammatory state, as indicated by NLR, in a Spanish population over 50 years of age. Furthermore, the study reported that better diet quality, assessed using the Spanish Healthy Eating Score, was significantly associated with reduced systemic inflammation [9]. Aydin et al. revealed that NLR and CRP levels were higher in children with obesity compared to healthy controls, whereas PLR levels were not significantly different between these two groups [10]. Similarly, in our study. NLR was significantly higher in the obesity and overweight groups compared to normal weight individuals (*p* = 0.001), while PLR did not show a statistically significant difference across BMI categories.

The NRI is a widely used tool for assessing nutritional status, particularly in clinical settings, and is calculated using serum albumin levels. Since albumin is a negative acute-phase reactant. Its synthesis is influenced not only by nutritional status but also by systemic inflammation. In individuals with obesity, despite a high BMI, protein–energy malnutrition may still occur—a condition referred to as “sarcopenic obesity” [26]. In patients with sarcopenic obesity, inadequate protein and calorie intake, together with chronic low-grade inflammation, may jointly contribute to reduced albumin levels despite excess body weight. While BMI tends to correlate positively with systemic inflammatory markers, NRI may correlate inversely, largely due to the inflammatory suppression of albumin synthesis and alterations in body composition [27]. Therefore, NRI may provide complementary insights to BMI in evaluating the complex relationship between nutritional status and inflammation in populations with obesity. In our study, although the NRI score increased significantly with BMI (*p* < 0.001), it remained ≥100 across all BMI categories, indicating no apparent nutritional risk in any group (Table 2). In addition, no significant correlations were found between NRI and systemic inflammation markers in the overweight group (Table 3), which may be consistent with the possibility of sarcopenic obesity. In individuals with sarcopenia, NRI may provide a falsely reassuring assessment of nutritional status, particularly in cases of sarcopenic obesity, where reduced muscle mass coexists with increased fat mass [28].

The HALP and PNI scores did not differ significantly across BMI groups, and CONUT scores were <1 in all BMI groups, indicating a normal nutritional status across the study population. These findings highlight that higher weight may not equate to better nutritional quality [12,13]. However, correlation analysis provided additional insights into the present study. In both overweight individuals and those with obesity, HALP scores showed inverse correlations with SII, NLR, and PLR, suggesting that reduced nutritional status is associated with increased inflammation (Table 3 and Table 4). Among the markers, HALP stood out as a particularly consistent and sensitive integrative index, inversely associated with inflammation and positively with PNI, similar to findings in previous cancer and cardiovascular studies [14,15].

Initially proposed in oncology settings, the HALP score has shown prognostic value in several malignancies, including gastric, colorectal, and hepatocellular carcinomas [15,16]. More recently, its utility has been explored in non-cancer populations, such as patients with cardiovascular disease and chronic kidney disease [29,30]. Its simplicity—requiring only routinely available blood parameters—makes it particularly attractive for widespread clinical use. Despite growing interest, data on the HALP score in healthy individuals and its variation across BMI categories are limited. Given that obesity is characterized by a state of chronic low-grade inflammation and altered hematologic and nutritional parameters, evaluating HALP in relation to BMI may provide novel insights into the subclinical inflammatory–nutritional burden of excess weight. The HALP score may reflect the imbalance between nutritional status and inflammation in individuals with obesity. A low HALP score can serve as a significant indicator of underlying complications such as metabolic syndrome, insulin resistance, or sarcopenic obesity. Given its integrative nature, HALP may provide valuable insights into the interplay between nutritional deficiencies and chronic low-grade inflammation in the context of obesity.

### Strengths and Limitations of the Study

Our study makes a unique contribution in several ways. First, to our knowledge, it is among the few to examine the relationship between multiple composite nutritional indices (HALP, PNI, CONUT, NRI) and systemic immune–inflammatory markers (SII, NLR, PLR) in an adult population stratified by BMI. Unlike many prior studies that focused on hospitalized or chronically ill patients, our inclusion of individuals without known chronic diseases allows for a clearer evaluation of subclinical inflammatory and nutritional dynamics related to adiposity. Second, the use of a wide array of validated indices—both nutritional and inflammatory—provides a comprehensive and multidimensional assessment of immune–nutritional interactions in the context of increasing BMI. We believe that these contributions broaden the existing knowledge and lay an essential groundwork for future prospective studies involving individuals without known chronic diseases.

This study has several limitations. One limitation of our study is that a priori sample size estimation was not performed, as the retrospective design required the inclusion of all eligible patients within a specific time frame. The absence of formal sample size calculation may have limited the statistical power and the generalizability of subgroup findings—particularly in the obesity category where greater heterogeneity exists (e.g., sarcopenic obesity). In addition, the retrospective nature and relatively limited sample size precluded subgroup analyses with additional variables or the calculation of sample size estimates for each subgroup, which may have restricted the ability to detect more nuanced associations. Nevertheless, a post hoc power analysis for the SII values—representing the parameter with the lowest level of statistical significance—demonstrated a power of 81.7%. Thus, the current sample size is considered sufficient for the present stage. Future prospective studies with larger cohorts and predefined subgroup stratifications are needed to validate and expand upon our results.

The retrospective and cross-sectional design of this study precludes causal inferences. While exclusion criteria aimed to remove confounders such as chronic disease and medication use, subclinical inflammation or undiagnosed conditions may still have influenced the biomarker profiles. Although we included only individuals without known chronic diseases, the BMI interval of 18.5–39.99 kg/m^2^ covers distinct categories—normal weight, overweight, and obesity—with different health implications. According to WHO definitions, overweight and obesity do not represent a healthy weight status. Therefore, even though the participants were free of chronic disease, the inclusion of individuals with overweight and obesity may inherently reflect varying metabolic and inflammatory profiles. In addition, smoking status and alcohol consumption were obtained from medical records, which may be subject to reporting inaccuracies and potential misclassification. These factors should be considered when interpreting our findings.

Another limitation of our study is that BMI does not differentiate between visceral and non-visceral adiposity or account for variations in muscle mass. Body composition parameters such as fat mass, muscle mass, or waist-to-hip ratio were not directly measured. This limits the ability to distinguish between visceral adiposity and sarcopenia, both of which influence inflammation and nutritional markers independently of BMI. Dietary intake, physical activity, and socioeconomic status—potential confounders affecting both inflammation and nutritional status—were not assessed.

Finally, the retrospective design of our study may compromise internal validity, as data collection relied on existing records rather than standardized prospective assessments. Furthermore, the lack of subgroup analyses and the absence of certain variables, such as measures of visceral adiposity, muscle mass, and detailed lifestyle factors, may limit the external validity and generalizability of our findings.

## 5. Conclusions

In conclusion, our study demonstrates that higher BMI, even among individuals without known chronic diseases, is associated with increased levels of systemic immune–inflammatory markers. Among the nutritional indices examined, the HALP score appears to reflect the inverse relationship between nutrition and inflammation most effectively. This index may be useful for early identification of subclinical inflammation in overweight and/or obesity populations. Routine use of simple blood-based scores like HALP in clinical settings could support preventive strategies by identifying individuals at risk for inflammation-related complications before overt disease manifests in individuals with high BMI.

We recommend that future cohort studies incorporate body composition measurements and detailed assessments of dietary intake and physical activity, and be conducted with larger sample sizes with predefined subgroup analysis. Investigating the association between nutritional scores and systemic immune–inflammatory indices in obesity remains a promising area for further research.

## Figures and Tables

**Table 1:** The comparison of baseline characteristics and laboratory findings of the study population by BMI.

	Normal (n = 156)		Overweight (n = 71)		Obesity (n = 63)		*p* Value
	Mean ± SD	Median (Min–Max)	Mean ± SD	Median (Min–Max)	Mean ± SD	Median (Min–Max)	
**Age (years)**	39.91 ± 10.45	40 (21–65)	42.51 ± 10.72	43 (17–59)	45.56 ± 9.63	47 (26–69)	^a^ 0.001 **
**Waist circumference (cm)**	83.51 ± 9.89	84 (60–101)	92.73 ± 10.38	92 (72–119)	106.81 ± 9.60	107 (86–128)	^a^ 0.001 **
**WBC (/µL)**	7108.33 ± 1629.43	7000 (3400–12,300)	6964.23 ± 1574.45	6800 (3700–12,400)	7522.54 ± 1626.82	7500 (3550–10,900)	^a^ 0.114
**CRP (mg/L)**	1.98 ± 1.56	1.8 (0.1–7)	3.14 ± 4.03	2 (0.3–27)	4.19 ± 3.26	2.9 (0.4–16)	^b^ 0.001 **
**FBG (mg/dL)**	86.58 ± 9.88	85.7 (60–119)	89.23 ± 15.46	85 (64–130)	95.06 ± 16.89	93 (64–158)	^b^ 0.001 **
**HbA1c (%)**	5.52 ± 0.38	5.6 (4.6–6.3)	5.59 ± 0.40	5.6 (4.6–6.4)	5.83 ± 0.45	5.9 (4.6–6.5)	^a^ 0.001 **
**Insulin (mU/L)**	8.10 ± 3.72	7.1 (2.5–28)	13.61 ± 6.82	12.5 (2–50)	18.92 ± 11.98	17 (5.1–67)	^b^ 0.001 **
**HOMA-IR**	1.72 ± 0.78	1.6 (0.5–5.6)	3.02 ± 1.50	2.8 (0.4–8.1)	4.39 ± 2.46	4.3 (1–12.4)	^b^ 0.001 **
**Creatinine (mg/dL)**	0.75 ± 0.46	0.7 (0.4–6)	0.75 ± 0.15	0.7 (0.5–1.1)	0.73 ± 0.15	0.7 (0.5–1.1)	^b^ 0.628
**Urea (mg/dL)**	18.48 ± 7.93	17 (5.5–39)	26.63 ± 6.72	25 (16–44)	25.61 ± 7.18	24 (13–44)	^a^ 0.001 **
**Uric acid (mg/dL)**	4.62 ± 1.25	4.6 (1.9–7.7)	4.68 ± 1.16	4.5 (2.7–8.2)	5.38 ± 1.29	5.4 (2.2–8.2)	^a^ 0.001 **
**Total Protein (g/dL)**	7.17 ± 0.38	7.2 (6.3–8.2)	8.24 ± 8.53	7.3 (6–79)	7.26 ± 0.43	7.2 (6–8.4)	^b^ 0.148
**Albumin (g/dL)**	4.59 ± 0.33	4.6 (4–5.5)	4.58 ± 0.32	4.6 (3.8–5.3)	4.49 ± 0.37	4.6 (3.5–5.1)	^a^ 0.127
**Total Cholesterol (mg/dL)**	185.33 ± 31.69	181 (84.8–310)	205.12 ± 34.9	206.8 (128.6–288.6)	213.76 ± 34.81	214 (138.8–302.6)	^a^ 0.001 **
**Triglyceride (mg/dL)**	105.10 ± 106.53	93 (11–1290)	124.92 ± 77.46	108 (38–535)	173.54 ± 109.47	153 (11–628)	^b^ 0.001 **
**VLDL-C (mg/dL)**	21.02 ± 21.31	18.6 (2.2–258)	24.98 ± 15.49	21.6 (7.6–107)	34.71 ± 21.89	30.6 (2.2–125.6)	^b^ 0.001 **
**HDL-C (mg/dL)**	54.31 ± 13.99	50.2 (15–106)	54.22 ± 16.55	51 (29–106)	46.29 ± 9.30	45 (32–68)	^a^ 0.001 **
**LDL-C (mg/dL)**	106.79 ± 28.95	101.8 (0–236)	125.92 ± 27.61	130 (69–189)	132.76 ± 25.76	138 (77–188)	^a^ 0.001 **

CRP: *C*-reactive Protein, FBG: Fasting Blood Glucose, HbA1c: Hemoglobin A1c, HDL-C: High-Density Lipoprotein Cholesterol, HOMA-IR: Homeostasis model assessment for insulin resistance, LDL-C: Low-Density Lipoprotein Cholesterol, VLDL-C: Very-Low-Density Lipoprotein Cholesterol, WBC: White Blood Cells. ^a^ One-Way Anova Test and Bonferroni Test, ^b^ Kruskal–Wallis Test and Dunn Bonferroni Test; ** *p* < 0.01. Bolded values denote statistically significant results.

**Table 2:** The association of BMI with nutrition scores and systemic immune–inflammatory indices.

	Normal (n = 156)		Overweight (n = 71)		Obesity (n = 63)		*p* Value
	Mean ± SD	Median (Min–Max)	Mean ± SD	Median (Min–Max)	Mean ± SD	Median (Min–Max)	
**PNI**	58.18 ± 0.69	58 (6.5–74.2)	56.33 ± 0.90	57 (46.5–73)	56.48 ± 1.00	58 (45.5–65.5)	^c^ 0.18
**NRI**	114.05 ± 0.50	114.33 (104.48–128.12)	123.17 ± 0.74	123.21(111.64–137.82)	135.04 ± 0.79	134.49 (107.66–155.62)	^c^ 0.001 **
**CONUT**	0.63 ± 0.05	1 (0–3)	0.48 ± 0.08	0 (0–3)	0.32 ± 0.90	0 (0–4)	^c^ 0.01 *
**SII (×100)**	441.33 ± 24.48	420.8 (44.1–1820.2)	617.82 ± 36.28	503.9 (157.6–2356)	569.59 ± 38.52	526.2 (189.3–1663.2)	^c^ 0.001 **
**PLR**	126.05 ± 3.97	122.9 (17.6–388.1)	139.21 ± 5.89	124.1 (64.2–380)	126.47 ± 6.26	119.5 (59.2–308)	^c^ 0.16
**NLR**	1.55 ± 0.07	1.4 (0.2–5.6)	2.13 ± 0.11	1.7 (0.8–7)	2.01 ± 0.11	1.8 (0.7–5.4)	^c^ 0.001 **
**HALP**	55.15 ± 4.46	50.21 (13.22–370.01)	67.24 ± 6.55	50.71 (19.99–628.33)	54.17 ± 6.95	49.39 (17.92–112.56)	^c^ 0.261

CONUT: Controlling Nutritional Status. HALP: Hemoglobin. Albumin. Lymphocyte. And Platelet Score. NLR: Neutrophil Lymphocyte Ratio. NRI: Nutritional Risk Index. PLR: Platelet Lymphocyte Ratio. PNI: Prognostic Nutritional Index. SII: Systemic Immune–Inflammation Index. ^c^ ANCOVA Test and Bonferroni Test; ** *p* < 0.01, * *p* < 0.05. Bolded values denote statistically significant results.

**Table 3:** The correlation of nutrition status with systemic immune–inflammatory indices in overweight participants.

	SII		PLR		NLR		PNI		HALP		NRI	
	r	*p*	r	*p*	r	*p*	r	*p*	r	*p*	r	*p*
**PLR**	0.837	<0.001 ***	-	-								
**NLR**	0.844	<0.001 ***	0.589	<0.001 **	-	-						
**PNI**	−0.295	0.013 *	−0.392	0.001 **	−0.462	<0.001 ***	-	-				
**HALP**	−0.23	0.046 *	−0.307	0.01 *	−0.170	0.160	0.342	0.004 **	-	-		
**NRI**	−0.101	0.403	0.069	0.571	−0.149	0.219	−0.144	**0.235**	−0.226	0.06	-	-
**CONUT**	0.307	0.01 *	0.302	0.011 *	0.449	<0.001 ***	−0.292	0.014 *	−0.112	0.355	−0.096	0.432

CONUT: Controlling Nutritional Status, HALP: Hemoglobin, Albumin, Lymphocyte, and Platelet Score, NLR: Neutrophil Lymphocyte Ratio, NRI: Nutritional Risk Index, PLR: Platelet Lymphocyte Ratio, PNI: Prognostic Nutritional Index, SII: Systemic Immune–Inflammation Index, Partial Correlation test; *** *p* < 0.001, ** *p* < 0.01, and * *p* < 0.05. Bolded values denote statistically significant results.

**Table 4:** The correlation of nutrition status with systemic immune–inflammatory indices in participants with obesity.

	SII		PLR		NLR		PNI		HALP		NRI	
	r	*p*	r	*p*	r	*p*	r	*p*	r	*p*	r	*p*
**PLR**	0.6	<0.001 ***	-	-								
**NLR**	0.887	<0.001 ***	0.371	0.003 **	-	-						
**PNI**	−0.337	0.007 **	−0.363	0.004 **	−0.498	<0.001 ***	-	-				
**HALP**	−0.522	<0.001 ***	−0.809	<0.001 ***	−0.358	0.004 **	0.601	<0.001 ***	-	-		
**NRI**	−0.162	0.209	0.183	0.155	−0.299	0.018 *	−0.062	0.632	−0.256	0.045 *	-	-
**CONUT**	−0.014	0.912	0.378	0.002 **	0.048	0.713	−0.166	0.198	−0.236	0.064	0.057	0.658

CONUT: Controlling Nutritional Status. HALP: Hemoglobin. Albumin. Lymphocyte and Platelet Score. NLR: Neutrophil Lymphocyte Ratio. NRI: Nutritional Risk Index. PLR: Platelet Lymphocyte Ratio. PNI: Prognostic Nutritional Index. SII: Systemic Immune–Inflammation Index. Partial Correlation test; *** *p* < 0.001, ** *p* < 0.01, and * *p* < 0.05. Bolded values denote statistically significant results.

## Data Availability

The original contributions presented in this study are included in the article. Further inquiries can be directed to the corresponding author.

## Abbreviations

BMI	Body Mass Index
CBC	Complete Blood Count
CONUT	Controlling Nutritional Status
CRP	*C*-reactive protein
ECLIA	Electrochemiluminescence Immunoassay
FBG	Fasting Blood Glucose
HALP	Hemoglobin, Albumin, Lymphocyte and Platelet
HbA1c	Hemoglobin A1c (HbA1c)
HDL	Low-Density Lipoprotein Cholesterol
HOMA-IR	Homeostasis Model Assessment for Insulin Resistance
HPLC	High-Performance Liquid Chromatography
IL-6	Interleukin-6
LDL-C	Low-Density Lipoprotein Cholesterol
NLR	Neutrophil–Lymphocyte Ratio
NRI	Nutritional Risk Index
PLR	Platelet–Lymphocyte Ratio
PNI	Prognostic Nutritional Index
SII	Systemic Immune–Inflammation Index
TNF-α	Tumor Necrosis Factor-α
VLDL-C	Very-Low-Density Lipoprotein Cholesterol
WBCs	White Blood Cells
